# A Multicomponent mHealth-Based Intervention (SWAP IT) to Decrease the Consumption of Discretionary Foods Packed in School Lunchboxes: Type I Effectiveness–Implementation Hybrid Cluster Randomized Controlled Trial

**DOI:** 10.2196/25256

**Published:** 2021-06-24

**Authors:** Rachel Sutherland, Alison Brown, Nicole Nathan, Serene Yoong, Lisa Janssen, Amelia Chooi, Nayerra Hudson, John Wiggers, Nicola Kerr, Nicole Evans, Karen Gillham, Christopher Oldmeadow, Andrew Searles, Penny Reeves, Marc Davies, Kathryn Reilly, Brad Cohen, Luke Wolfenden

**Affiliations:** 1 Hunter New England Population Health Wallsend Australia; 2 School of Medicine and Public Health University of Newcastle Callaghan Australia; 3 Hunter Medical Research Institute New Lambton Heights Australia; 4 Priority Research Centre for Health Behaviour University of Newcastle Callaghan Australia; 5 Department of Nursing and Allied Health School of Health Sciences Swinburne University of Technology Hawthorn Australia; 6 Mid North Coast Local Health District Port Macquarie Australia; 7 Central Coast Local Health District Gosford Australia; 8 New South Wales Ministry of Health Liverpool Australia; 9 SkoolBag Sydney Australia

**Keywords:** childhood obesity, lunchboxes, children, child nutrition, mHealth, schools, hybrid, randomized controlled trial, technology

## Abstract

**Background:**

There is significant opportunity to improve the nutritional quality of foods packed in children’s school lunchboxes. Interventions that are effective and scalable targeting the school and home environment are therefore warranted.

**Objective:**

This study aimed to assess the effectiveness of a multicomponent, mobile health–based intervention, SWAP IT, in reducing the energy contribution of discretionary (ie, less healthy) foods and drinks packed for children to consume at school.

**Methods:**

A type I effectiveness–implementation hybrid cluster randomized controlled trial was conducted in 32 primary schools located across 3 local health districts in New South Wales, Australia, to compare the effects of a 6-month intervention targeting foods packed in children’s lunchboxes with those of a usual care control. Primary schools were eligible if they were not participating in other nutrition studies and used the required school communication app. The Behaviour Change Wheel was used to co-design the multicomponent SWAP IT intervention, which consisted of the following: school lunchbox nutrition guidelines, curriculum lessons, information pushed to parents digitally via an existing school communication app, and additional parent resources to address common barriers to packing healthy lunchboxes. The primary outcome, mean energy (kilojoules) content of discretionary lunchbox foods and drinks packed in lunchboxes, was measured via observation using a validated school food checklist at baseline (May 2019) and at 6-month follow-up (October 2019). Additional secondary outcomes included mean lunchbox energy from discretionary foods consumed, mean total lunchbox energy packed and consumed, mean energy content of core lunchbox foods packed and consumed, and percentage of lunchbox energy from discretionary and core foods, all of which were also measured via observation using a validated school food checklist. Measures of school engagement, consumption of discretionary foods outside of school hours, and lunchbox cost were also collected at baseline and at 6-month follow-up. Data were analyzed via hierarchical linear regression models, with controlling for clustering, socioeconomic status, and remoteness.

**Results:**

A total of 3022 (3022/7212, 41.90%) students consented to participate in the evaluation (mean age 7.8 years; 1487/3022, 49.22% girls). There were significant reductions between the intervention and control groups in the primary trial outcome, mean energy (kilojoules) content of discretionary foods packed in lunchboxes (–117.26 kJ; 95% CI –195.59 to –39.83; *P*=.003). Relative to the control, the intervention also significantly reduced secondary outcomes regarding the mean total lunchbox energy (kilojoules) packed (–88.38 kJ; 95% CI –172.84 to –3.92; *P*=.04) and consumed (–117.17 kJ; 95% CI –233.72 to –0.62; *P*=.05). There was no significant difference between groups in measures of student engagement, consumption of discretionary foods outside of school hours, or cost of foods packed in children’s lunchboxes.

**Conclusions:**

The SWAP IT intervention was effective in reducing the energy content of foods packed for and consumed by primary school–aged children at school. Dissemination of the SWAP IT program at a population level has the potential to influence a significant proportion of primary school–aged children, impacting weight status and associated health care costs.

**Trial Registration:**

Australian Clinical Trials Registry ACTRN12618001731280; https://www.anzctr.org.au/Trial/Registration/TrialReview.aspx?id=376191&isReview=true

**International Registered Report Identifier (IRRID):**

RR2-10.1186/s12889-019-7725-x

## Introduction

Preventing the onset of overweight and obese status in children is a global public health priority [1], given that it impacts negatively on physical health, psychological well-being, and long-term chronic disease risk [2]. The frequent overconsumption of energy-dense, nutrient-poor, or “discretionary” foods throughout childhood, which displace the consumption of core foods consistent with dietary guidelines, is known to be a major contributor to the development of being overweight and obese [3]. Of concern, the poor dietary patterns that are established in childhood track into adulthood and increase the risk of adults being overweight or obese [4]. To address this, the World Health Organization (WHO) recommends implementing populationwide interventions to support the establishment of eating habits in children that are consistent with dietary guidelines [5].

Children consume up to two-thirds of their daily energy intake at school [6]. Consequently, schools have been identified as an optimal setting to implement public health nutrition interventions [5]. Internationally, school-based nutrition research has focused on improving the provision or sale of foods at school canteens [7] or cafeterias [8]. However, in many countries, such as Australia [9], the United Kingdom [10], New Zealand [11], and Denmark [12], a significant proportion of children consume food brought to school from home in a lunchbox. Further, research suggests that the nutritional quality of foods packed in school lunchboxes may be poorer than that available at or provided by schools. For example, in Australia, approximately 5% of items sold at school canteens are discretionary items [13] compared to 40% in children’s lunchboxes [14]. A cross-sectional study undertaken in Australia of 1681 students found that lunchboxes contain an average of 3.1 servings of discretionary foods (1200 kJ) and contributed to over 3000 kJ, which is significantly higher than that recommended in dietary guidelines [15]. A further Australian study involving 2143 primary school aged children (mean age 7.96 years) found that just 12% of students’ lunchboxes contain only core foods (ie, minimally processed foods recommended in Australian Dietary Guidelines), with a quarter containing 4 or more discretionary servings [16], exceeding the maximum daily amount for children of this age. Similar nutrient compositions have been observed in lunchboxes across the globe, including in New Zealand [11], the United Kingdom [10,17], Canada [18], and the United States [19].

Current evidence regarding the effectiveness of school lunchbox interventions is equivocal. A recent systematic review of such interventions in the school and childcare setting identified just 10 trials and suggested they had little to no effect on the nutritional quality of foods packed or consumed by students [20]. Existing interventions have employed either passive information dissemination strategies to parents, which have limited reach and engagement, or have used intensive face-to-face group-based strategies attracting a biased population group and presenting considerable challenges to implement at scale.

Mobile text messaging– and mobile app–based interventions have been proven to be a scalable and effective approach for improving a variety of health behaviors—including those of parents—to provide a better child diet [21,22]. Our previous pilot study in 12 schools, assessing the feasibility, acceptability, and potential efficacy of the multicomponent SWAP IT intervention [16], used an existing school mobile communication app, along with newly developed school nutrition guidelines, school curriculum, and resources for parents, to encourage a “swap” in their children’s lunchboxes of discretionary foods to healthier alternatives consistent with the Australian Dietary Guidelines (“everyday“ foods) [23]. The intervention approach was found to be highly feasible to deliver and acceptable to both schools and parents, demonstrating promising short-term improvements in the nutritional quality of foods packed in lunchboxes [16]. Following the encouraging findings of the pilot study, our primary aim was to conduct an adequately powered randomized trial to assess the effectiveness of the SWAP IT multicomponent lunchbox intervention in reducing the kilojoule content from discretionary foods and drinks both packed and consumed by children from school lunchboxes while at school relative to usual care. We also sought to evaluate the effectiveness of the intervention on a range of secondary outcomes, including mean lunchbox energy from discretionary foods consumed, mean total lunchbox energy packed and consumed, mean energy content of core lunchbox foods packed and consumed, percentage of lunchbox energy from discretionary and core foods, measures of school engagement, consumption of discretionary foods outside of school hours, and lunchbox cost.

## Methods

### Ethics and Registration

The research was conducted and reported in accordance with the requirements of the Consolidated Standards of Reporting Trials (CONSORT) statement [24]. Approval to conduct this study was obtained from the Hunter New England Human Research Ethics Committee (reference #06/07/26/4.04), University of Newcastle (reference #H-2008-0343) and the New South Wales (NSW) State Education Research Applications Process (#2018247) and was prospectively registered with Australian New Zealand Clinical Trials Register (#12618001731280). A detailed description of the methods and intervention are outlined in the study protocol [25].

### Study Design and Setting

**Figure 1:**
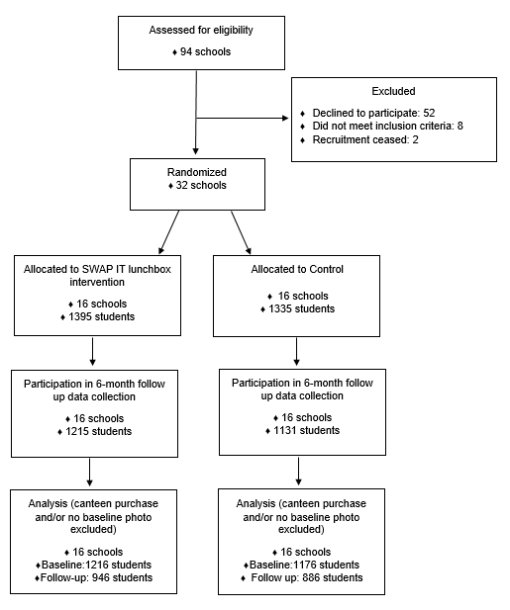
Consolidated Standards of Reporting Trials (CONSORT) flow diagram.

A type I effectiveness–implementation hybrid cluster randomized controlled trial was conducted with 32 primary (students aged approximately 5-12 years) schools across 3 local health districts in NSW, Australia (Figure 1). Schools were randomized to receive a 6-month (2 school terms), multicomponent lunchbox intervention or a usual care control arm (16 schools per arm). Outcome assessments were conducted in a cohort of students at baseline and at 6 months after randomization. The primary outcome was mean energy (kilojoules) content of discretionary lunchbox foods and drinks packed in lunchboxes assessed via lunchbox observation. Other registered outcomes related to implementation processes, including intervention acceptability, appropriateness and feasibility [26,27], cost-effectiveness of the intervention, and impact on mean daily nutrient consumption, will be reported separately.

### Sample and Participants

#### Schools

Schools were considered eligible if they met the following criteria: government primary schools catering for students from kindergarten to year 6 and located in one of the participating local health districts, greater than 120 student enrolments, current users of the preferred school mobile communication app (SkoolBag), and not participating in other nutrition-based research studies. Schools purchase the communication app for a nominal fee annually, which is then free for parents to download, enabling direct school–parent communication. The app is used by approximately 60% of schools in the region. Central schools (catering for students aged 5-18 years) and schools primarily catering for children with additional needs (such as intellectual disabilities) were excluded. According to a random number generator in Excel (Microsoft Corporation), eligible schools meeting the above criteria were sent a letter of invitation in random order. One week following the invitation, a member of the research team contacted the school principal via telephone to seek consent. A face-to-face meeting was offered to all schools to outline the requirements of the study. Recruitment and consent of schools occurred between February 2019 and May 2019. Recruitment continued until 32 schools provided active signed principal consent to participate.

#### Parents and Students

Opt-in parental consent was required for children and parents to participate in the evaluation of the behavioral outcomes. Parents were also required to be active users of the school communication app, defined as downloading the school communication app on the parent consent form. A strategy to recruit parents and students was developed based on the pilot study and reviews of evidence for facilitating participation in school-based research [16,28]. Following principal consent, all parents with a child enrolled in classes from kindergarten to year 6 (5-12 years) were invited to participate in the study evaluation measures, which included a lunchbox observational assessment, parent survey, and student survey (year 5 and 6 students). Students were provided with an information package outlining the study and a consent form. Parents were asked via the consent form if they were an active user (ie, downloaded the app) of the school communication app. One week after the information package was distributed, parents who had not returned a consent form were telephoned by school-employed staff. A replacement consent form was distributed via mail to parents who provided verbal consent over the phone.

### Randomization and Blinding

Following baseline data collection, schools (cluster) were randomly allocated in a 1:1 ratio to the intervention or control group based on a random number function in Excel. Randomization was undertaken by a statistician not involved in contacting schools in the study intervention or assessment and stratified by the socioeconomic status of school locality using the Socio-Economic Indexes for Areas (SEIFA 2016), as socioeconomic status is associated with lunchbox contents and child diet [29,30]. Research personnel involved in data collection and lunchbox content analysis were blind to group allocation, as all identifiable school information was removed prior to data analysis. Data collection staff were not informed of group allocation; however, this might have been disclosed to them by school staff during field activity. Due to the inability to conceal intervention delivery, school personnel were notified of their group allocation via a phone call.

### Multicomponent Intervention

The multicomponent intervention based on the previous pilot was codeveloped by a multidisciplinary team comprising academic and end-user stakeholders from government health agencies, educational systems, universities, and technology partners and included parent representatives with expertise in nutrition, school-based health interventions, behavior change, implementation science, and technology-based interventions.

### Conceptual Framework

The Behaviour Change Wheel [31] was used to guide the development of the intervention. Extensive formative research encompassing a review of published literature; focus groups with parents to identify local contextual barriers; telephone interviews with parents (n=228) (L Janssen, R Sutherland, and N Nathan; unpublished data, 2019) and principals (n=196) [32] to assess barriers, acceptability of intervention strategies, and content and delivery mode; and a literature review of existing lunchbox interventions [20] were undertaken to select behavior change techniques and strategies to support parents to pack healthy school lunchboxes. Multimedia Appendix 1 outlines the Behaviour Change Wheel mapping process and outlines the chosen behavior change techniques incorporated into the SWAP IT intervention.

**Figure 2:**
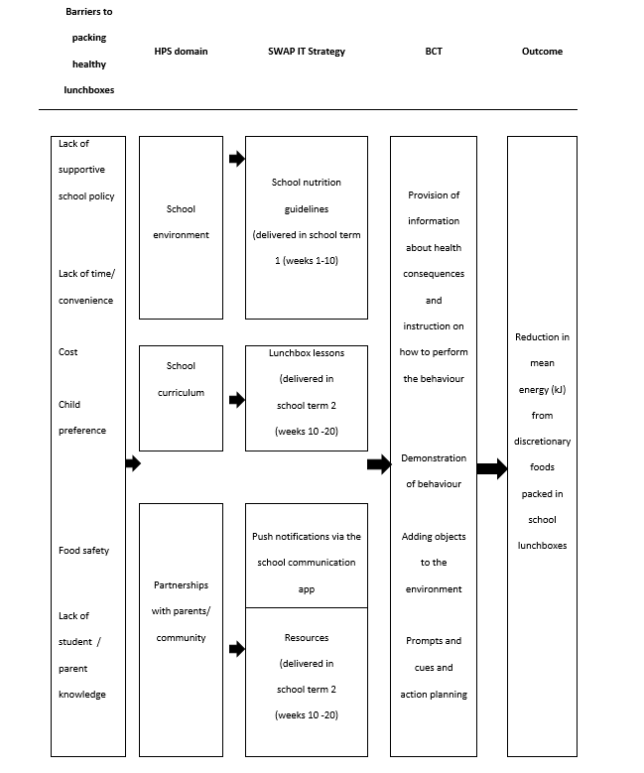
SWAP IT logic model. BCT: behaviour change technique; HPS: health promoting schools framework.

Figure 2 provides an overview of the SWAP IT intervention logic. The SWAP IT intervention encouraged lunchbox “swaps” from discretionary food items to Australian Dietary Guideline–based healthier alternatives known as “everyday” foods. The multicomponent lunchbox intervention consisted of 4 strategies outlined in the following section. The mobile health component included weekly pushed messages to parents delivered via an existing school mobile communication app, SkoolBag, in addition to embedding lunchbox content within the app for parents to access. A detailed 4-part description of the intervention has been published in a protocol [25], and the intervention included the 4 following strategies:

1. Lunchbox nutrition guidelines: Using a template developed by the project team, school principals developed, endorsed, and disseminated nutrition guidelines to parents which were consistent with the WHO and the NSW Department of Education Nutrition in Schools policy [33]. Guidelines were disseminated to parents in the first 5 weeks of the intervention via the SkoolBag app and school newsletters to demonstrate schools’ endorsement of the SWAP-IT program.
2. Weekly pushed lunchbox messages: Through the SkoolBag app, 10 weekly electronic messages (push notifications) to support the packing of healthy lunchboxes were disseminated to parents or caregivers. Messages were codeveloped by the research team, public health nutritionists, health promotion practitioners, teachers, and parents and were optimized and refined via a study involving 511 parents [34]. The distribution of the messages via the school communication app was managed centrally by the project team. This allowed all parents at the schools allocated to the intervention group who had downloaded the app to receive the pushed messages via the research team and prevented the need to rely on each individual school to push the weekly content to parents. This centrally coordinated effort therefore did not require school time or resources and thereby maximized the fidelity of the intervention. The pushed messages aligned to parent-reported barriers to packing healthy school lunchboxes: lack of time or convenience, knowledge of suitable swaps, child preference, cost, food safety, and lack of school nutrition policy. Where possible, a swap within the same food category was suggested (eg, for packaged foods). The pushed messages were designed to act as prompts and cues to reinforce packing of everyday foods. The messages were connected to embedded videos developed by the research team to align to parent-reported barriers which provided tips and suggestions to assist parents to pack everyday foods that were quick, convenient, and low cost and to connect parents with tools and resources to improve their knowledge and skills to swap out discretionary foods and pack everyday foods.
3. Resources for parents: Links embedded in the app messages connected parents with electronic resources housed on the program website. These resources provided information regarding health consequences, simple healthy lunchbox swaps that addressed child preference, cost, convenience, and food safety. Physical resources, including a SWAP IT ideas booklet (lunchbox ideas), clear drink bottle for water, and an ice brick to support food safety, were also provided to parents and were distributed to students and parents via the schools’ usual methods of dissemination.
4. Curriculum resources for schools: Schools were provided with a short online teacher professional learning module (10 minutes) developed by the research team, which included public health nutritionists, health promotion practitioners, and teachers outlining the rationale for the study and providing the skills and resources required to deliver the classroom curriculum lessons. Schools were also provided stage-appropriate curriculum resources which were codeveloped by the research team with input from teachers, parents, and education partners to align with syllabus outcomes that were developed by dietitians and teachers in order to reinforce healthy food preferences. This required teachers to deliver 3 curriculum lessons 10 minutes in duration. Curriculum resources were designed to address the identified barrier to packing a healthy lunchbox of “child preference for discretionary foods.”

### Control Schools

Schools allocated to the control group had access to the SkoolBag app but not the lunchbox intervention content. The SWAP IT website was freely accessible by the general public, including parents and schools; however, schools and parents were not notified or directed to this site. There was no information (nutrition or otherwise) provided to the control group, and they participated in data collection only and continued usual school business.

### Data Collection and Measures

#### Lunchbox Energy

The primary outcome was the mean energy (kilojoules) content of discretionary foods packed in the school lunchboxes by parents who were users of the school mobile app, assessed at baseline and at 6-month follow-up. A detailed description of the study measures and data collection methods have been described in a published protocol [25]. Lunchbox energy content was assessed from photos of lunchboxes taken at school by trained research assistants prior to the first meal break with a valid and reliable lunchbox observational audit, known as the School Food Checklist (SFC) [35,36]. The SFC is a previously validated tool shown to be accurate and reliable in measuring energy from food and drinks for the Australian context. The SFC [35,36] enabled the assessment of the kilojoule content and serving size for each lunchbox item. Two trained dietitians observed each school lunchbox photo and classified each food and drink item according to its SFC category as “everyday foods” or “discretionary foods” and assessed the kilojoule content and serving size for each lunchbox item and the serving size. The checklist included 20 food and drink categories, including main food items, such as bread, fast food, and leftovers/mixed dishes; and snack items such as noodles, packaged snacks, biscuits and crackers, chocolate and candy, cheese, eggs, dried fruit and nuts, muesli and fruit bars, cakes and buns, muffins and scones, pastries, desserts, yoghurt, fruit, vegetables, milk, soft drink, and water and fruit juice. “Everyday” items referred to food and drink items that were part of the core food groups as determined by the Australian Dietary Guidelines [23]. Food items classified as “discretionary” were items considered to be energy dense with minimal nutritional value, including cakes, chocolate, candy, chips, muesli bars, and fast food [23]. The serving size of each lunchbox item and kilojoules per serving information was obtained from FoodWorks Professional Edition V7 (version 7, Xyris Software). To further aid this process, decision rules developed in the previous study [16] were used to ensure standardization of assessments.

The secondary outcomes associated with lunchbox energy were mean total energy (kilojoule) packed within the lunchbox; mean total energy (kilojoules) consumed from the lunchbox; mean energy (kilojoules) from discretionary foods and drinks consumed within the lunchbox; mean energy (kilojoules) from healthy foods packed and consumed from the lunchbox; and percentage of lunchbox energy from discretionary and healthy foods and drinks, both packed and consumed. Data were collected at baseline and immediately after the 6-month intervention with the SFC as outlined in the previous section. Following the analysis of the premeal lunchbox photo, dietitians analyzed the postmeal photo.

#### Student Consumption of Discretionary Foods Outside of School Hours

At baseline and at follow-up, parents were asked to report, via a short telephone survey, on their child’s intake of discretionary foods outside of school hours and on weekends to identify any compensatory nutrition behavior occurring outside of school hours. Measures were taken from the NSW Schools Physical Activity and Nutrition Survey [37]. Parents reported on the following 6 categories of discretionary foods: (1) fried potato products, (2) potato chips and other salty snacks, (3) sweet biscuits and cakes, (4) confectionary, (5) ice cream or ice blocks, and (6) fruit juice. The frequency of consumption for consenting students was reported at baseline and immediately after the intervention at 6 months.

#### Procedures

To assess the foods packed in the lunchbox (premeal assessment) on a randomly selected school day prior to recess, at lunch, or during in-class vegetable and fruit breaks [38,39], consenting students were asked to display the contents of their lunchbox on their desk in the classroom. Parents and students were not informed of the exact day of data collection. A preprepared paper grid was placed under the lunchbox contents and used to assess the scale and serving size of the items. Any foods not easily identified were discussed with the student and further details were recorded on the grid paper prior to being photographed. The photo was taken by trained research assistants prior to any foods being consumed. Students were asked if they intended to purchase food from the canteen that day, and if so, were removed from the analysis.

To assess the consumption of foods packed in the lunchbox (postmeal assessment), on the same day, students were asked to keep all unconsumed or partially consumed food items in their lunchboxes. Following all meal breaks, students were asked to place unconsumed or partially consumed items from their lunchbox onto the grid paper, and a second photograph of all remaining food was taken. Measures relating to consumption were based on the second photograph of the day being taken after all meal breaks had occurred and all uneaten food had been placed back into the lunchbox container. Consumption was calculated by subtracting the postmeal assessment from the premeal assessment.

Trained dietitians, blinded to group allocation, observed each school lunchbox photo in order to classify each food and drink item according to its SFC category and the serving size. All lunchbox photos were assessed by 2 dietitians working together to make a consensus decision on the analysis for each lunchbox. To further aid this process, decision rules were developed to ensure standardization of assessments. Differences in opinion between dietitians were resolved following consultation with a third dietitian assessor. Following the analysis of the premeal lunchbox photo, dietitians then analyzed the postmeal photo. Energy consumption was calculated by subtracting the energy content of foods and drinks remaining in students’ lunchboxes at the postmeal assessment from the energy content of foods and drinks in the lunchbox during premeal assessment (“foods consumed”).

#### Student School Engagement

We also assessed impact on engagement, as research suggests that improved nutrition correlates with greater school attendance, improved concentration, and higher academic achievement [40]. At baseline and at follow-up, students in years 5 and 6 completed selected items from the validated School Engagement Measure via a pen and paper survey. The School Engagement Measure is a 19-item survey that provides a measure of students’ behavioural (5 items), emotional (6 items), and cognitive engagement (8 items) at school, which are outcomes considered important for achieving positive academic outcomes [41].

#### Student Consumption of Discretionary Foods Outside of School Hours

To ensure any reduction in energy intake occurring while at school did not result in compensatory intake outside of school hours (potential adverse event), parents were asked via a short telephone survey at baseline and at follow-up to report on their eldest eligible child’s intake of discretionary foods outside of school hours and on weekends. Measures were taken from the NSW Schools Physical Activity and Nutrition Survey [37]. Parents reported on 6 categories of discretionary foods, including (1) fried potato products, (2) potato chips and other salty snacks, (3) sweet biscuits and cakes, (4) confectionary, (5) ice cream or ice blocks, and (6) fruit juice and reported the frequency of consumption as never or rarely, 1 to 2 times per week, 3 to 4 times per week, 5 to 6 times per week, once per day, or 2 or more times per day.

#### Lunchbox Cost

It has been hypothesized that one potential adverse effect of encouraging healthier lunchbox swaps is increased family financial burden due to the potential higher cost of healthier products [42]. To assess this, the mean cost of lunchbox items before and after intervention was assessed via the SFC and were compared between intervention and control groups at baseline and at follow-up to determine if the intervention resulted in any adverse financial effects for families. Costing was determined with an average of prices from foods within the category accessed from a local retail audit of similar foods as of October 2018.

### Statistical Analysis

Analyses were conducted using SAS version 9.3 (SAS Institute) from January 2020 to June 2020. School and student characteristics were summarized for intervention and control schools. Summary statistics are used to describe all variables of interest. Students that resided in postcodes ranked in the top 50% of state postcodes based on the 2016 SEIFA [30] were categorized into “higher socioeconomic areas,” whereas those in the lower 50% were categorized into “lower socioeconomic areas.” Students’ postcodes were also used to categorize their locality as either “rural” (those schools in outer regional, remote, or very remote areas) or “urban” (those in regional or major cities) based upon the 2016 Accessibility/Remoteness Index of Australia [43].

The differences between groups in the primary and secondary outcomes were assessed using hierarchical linear (or logistic for binary outcomes) regression models. Models were adjusted for SEIFA, remoteness, and baseline values, and a random level intercept for schools was included to adjust for the clustered design of the study. Analysis followed intention-to-treat principles, where schools and students were analyzed according to their randomized treatment allocation. All statistical tests were 2-tailed with an α of .05. As specified in the study protocol [25], data were analyzed only for students whose parents had reported downloading the required SkoolBag app to ensure exposure to the intervention, and students intending to purchase food or drinks from the canteen or who did not bring lunch were removed from the primary analysis to focus on students whose lunchbox was their source of energy for the day [16].

### Sample Size and Power

According to our pilot results [16], a standard lunchbox contains 1089 kJ (SD 900 kJ) of discretionary foods. With an intraclass correlation coefficient of 0.05, 32 schools with 140 students per school enabled detection of a 200-kJ difference between groups at follow-up on the primary trial outcome, with 80% power at a significance level of *P*<.05. As approximately 420 kJ across a whole day has the potential to reduce the prevalence of childhood obesity [44,45] and as it is recommended that a child consumes a third of their daily energy requirements while at school [9], this magnitude of effect was considered meaningful at a population level.

## Results

### Sample

A sample of 94 schools was assessed for eligibility to participate in the study, and 91 were approached in order to obtain the quota of 32 consenting schools (35.2%). Consenting and nonconsenting schools were similar in geographic location, size, and school socioeconomic status, with the 32 consenting schools enrolling a total of 7212 students (or 5048 families). Of these, 3022 provided parental consent to participate in the lunchbox observation to evaluate the outcomes of the study (41.90%). From the 3022 consenting students, 2730 (1395 intervention and 1335 control) lunchboxes were observed at baseline and 2346 (1215 intervention, 1131 control) at follow-up, with the discrepancy being due to student absences and school events or excursions. Table 1 outlines the school and student characteristics of those consenting to participate. At baseline, the consenting schools and students allocated to the intervention and control groups had similar characteristics; however, the intervention group had a higher proportion of schools located in disadvantaged areas.

**Table 1:** Sample characteristics of schools and students at baseline.

Characteristics				Intervention		Control
**Schools**						
	Allocation, n			16		16
	**Location, n (%)**					
		Urban	8 (50.0)		6 (37.5)	
		Rural	8 (50.0)		10 (62.5)	
	**Socioeconomic status^a^, n (%)**					
		Most disadvantaged	13 (81.2)		13 (81.2)	
		Least disadvantaged	3 (18.8)		3 (18.8)	
	Schools with greater than 10% Aboriginal or Torres Strait Islander student enrolments, n			10		11
**Students**						
	Allocation, n			1216		1176
	**Sex, n (%)^b^**					
		Female	592 (50.04)		550 (48.37)	
		Male	591 (49.96)		587 (51.63)	
	Mean age (years)			7.88		7.68
	**Socioeconomic status^a^, n (%)**					
		Most disadvantaged	938 (77.14)		789 (67.09)	
		Least disadvantaged	278 (22.86)		387 (32.91)	

^a^Socioeconomic status is based on SEIFA Index of relative socioeconomic disadvantage 2016: most disadvantaged = lowest quartiles of SEIFA; least disadvantaged = highest quartiles of SEIFA.

^b^Information on sex missing for 72 students.

### Primary Outcome: Mean Energy (Kilojoules) Content of Discretionary Foods Packed From the School Lunchboxes

At 6-month follow-up, the difference between the intervention and control group in the mean energy (kilojoules) content of discretionary foods packed in school lunchboxes was –117.71 kJ (95% CI –195.59 to –39.83; *P*=.003). A sensitivity analysis on the primary outcome using complete cases indicated a similar result of –120.43kJ (95% CI –200.82 to –40.04; *P*=.005).

### Secondary Lunchbox Energy Outcomes

The mean total energy (kilojoules) packed in lunchboxes (–88.38 kJ; 95% CI –172.84 to –3.92; *P*=.04) and mean total energy (kilojoules) consumed from lunchboxes (–117.17kJ; 95% CI –233.72 to –0.62; *P*=.05) both reduced in favor of the intervention group. There was also a significant reduction in percentage of lunchbox energy packed from discretionary foods between groups (–3.16%; 95% CI –5.46 to –0.86; *P*=.01), while the percentage of lunchbox energy from everyday foods increased (3.16%; 95% CI 0.86-5.46; *P*=.01). A significant reduction favoring the intervention group in the mean energy (kilojoules) from discretionary foods consumed from lunchboxes (–96.31kJ; 95% CI –194.63 to 2.01; *P*=.05) was also observed. There was no statistical difference between groups in the mean lunchbox energy from everyday foods (kilojoules) packed in lunchboxes (32.85 kJ; 95% CI –31.61 to 97.31; *P*=.31) or consumed (–21.91 kJ; 95% CI –112.38 to 68.56; *P*=.62). Table 2 outlines the lunchbox energy packed and consumed by group. Multimedia Appendix 2 outlines the food and drink items packed in lunchboxes.

**Table 2:** Mean energy and percentage of energy from everyday and discretionary foods packed and consumed from student lunchboxes.

Outcome		Intervention			Control			Difference in energy between groups at follow-up, mean (95% CI)		*P* value
		Baseline mean, (SD) (n=1216)	Follow-up, mean (SD) (n=946)	Baseline, mean (SD) (n=1176)		Follow-up, mean (SD) (n=886)				
**Daily energy (kilojoules) packed in student lunchboxes**										
	Primary outcome: lunchbox energy from discretionary foods packed in lunchboxes	1214.86 (876.49)	1156.77 (841.76)	1067.38 (898.82)		1105.06 (859.06)	–117.26 (–195.59 to 39.83)		.003	
	Lunchbox energy from everyday foods packed in lunchboxes	1616.19 (628.34)	1610.93 (624.41)	1644.17 (621.73)		1605.81 (610.02)	32.85 (–31.61 to 97.31)		.31	
	Total lunchbox energy packed in lunchboxes	2831.05 (927.81)	2767.70 (873.52)	2711.54 (962.33)		2710.87 (878.44)	–88.38 (–172.84 to –3.92)		.04	
**Daily lunchbox energy (kilojoules) consumed by students**										
	Lunchbox energy from discretionary foods consumed from lunchboxes	901.30 (745.60)	876.70 (717.23)	744.19 (717.20)		802.75 (677.23)	–96.31 (–194.63 to 2.01)		.05	
	Lunchbox energy from everyday foods consumed from lunchboxes	1270.85 (631.79)	1282.56 (622.95)	1304.69 (600.58)		1341.72 (607.53)	–21.91 (–112.38 to 68.56)		.62	
	Total lunchbox energy consumed from lunchboxes	2172.15 (895.82)	2159.26 (810.78)	2048.88 (853.84)		2144.48 (743.22)	–117.17 (–233.72 to –0.62)		.05	
**Lunchbox energy coming from discretionary and everyday foods (%)**										
	Packed lunchbox energy from discretionary foods	40.10 (23.31)	39.04 (23.94)	35.84 (23.69)		37.90 (23.81)	–3.16 (–5.46 to –0.86)		.01	
	Packed lunchbox energy from everyday foods	59.90 (23.31)	60.96 (23.94)	64.16 (23.69)		62.10 (23.81)	3.16 (0.86 to 5.46)		.01	
Total cost (Aus $) of lunchbox items		3.94 (1.35)	3.91 (1.36)	3.78 (1.38)		3.78 (1.32)	–0.06 (–0.18 to 0.07)		.37	

#### Student Engagement

Table 3 outlines the student engagement measures. There were no observed differences between groups for any measure of student engagement after the 6-month intervention, including for student total school engagement measure score (–0.08; 95% CI –0.18 to 0.02; *P*=.10), student behaviour (–0.05; 95% CI –0.15 to 0.04; *P*=.24), or emotional (–0.08; 95% CI –0.2 to 0.06; *P*=.26) or cognitive engagement (0.09; 95% CI –0.22 to 0.05; *P*=.20).

**Table 3:** Mean school engagement measure by group at baseline and at follow-up.

Mean school engagement score	Intervention			Control			Difference in engagement between groups at follow-up, mean (95% CI)		*P* value
	Baseline, mean (SD) (n=364)	Follow-up, mean (SD) (n=309)	Baseline, mean (SD) (n=299)		Follow-up, mean (SD) (n=241)				
Behavior score	4.12 (0.59)	4.09 (0.62)	4.11 (0.65)		4.14 (0.66)	–0.05 (–0.15 to 0.04)		.24	
Emotion score	3.55 (0.91)	3.33 (0.99)	3.56 (0.92)		3.40 (0.98)	–0.08 (–0.22 to 0.06)		.26	
Cognitive score	2.92 (0.87)	2.80 (0.87)	2.87 (0.83)		2.83 (0.85)	–0.09 (–0.22 to 0.05)		.20	
Total school engagement	3.44 (0.66)	3.31 (0.71)	3.42 (0.68)		3.35 (0.70)	–0.08 (–0.18 to 0.02)		.10	

#### Student Consumption of Discretionary Foods Outside of School Hours

There were no differences between groups in the foods consumed outside of school hours, indicating no compensatory consumption of discretionary foods outside of care.

#### Lunchbox Cost

The total cost of lunchbox foods following the intervention did not differ between groups (–Aus $0.06; 95% CI –0.18 to 0.07; *P*=.37; Table 2).

## Discussion

This trial investigated the effectiveness of the SWAP IT intervention on the energy of students’ lunchbox foods, both packed and consumed, using an existing school communication app provided directly to parents. Relative to lunchboxes in the control group, the lunchboxes in the intervention group contained significantly less mean energy from discretionary foods corresponding to 117 kJ per day or a 600 kJ reduction over a school week. The SWAP IT intervention also resulted in a reduction in mean energy from discretionary foods that were consumed by students (96.31 kJ). The mean total lunchbox energy both packed and consumed was also significantly less in intervention lunchboxes, and the percentage of energy from discretionary foods decreased by 3.16%, while percentage energy from everyday foods correspondingly increased. The lunchbox energy coming from everyday foods that were consistent with dietary guidelines did not statistically differ between groups, indicating the change in total energy observed was primarily from a reduction in discretionary foods. These favorable nutrition outcomes occurred while the cost of packing a lunchbox remained stable across groups, indicating the changes made to lunchboxes did not result in additional costs. The intervention, however, did not result in changes to student school engagement at school.

Although it is challenging to make direct comparisons, the magnitude of reduction in energy from discretionary foods appears favorable compared to previous lunchbox interventions. Of the 10 included studies within a systematic review of lunchbox interventions conducted within the school and childcare environment [20], 4 targeted the packing of discretionary foods, with evidence for the effectiveness of interventions on what is packed in lunchboxes in relation to discretionary foods, sugar-sweetened drinks, or other core foods being equivocal. Of the 2 studies conducted in the school environment, results were mixed, with interventions impacting on the reduction of either high fat salty snacks (reduction of 2.8 gm of savory snacks; *P*=.04) or sweet confectionary, fruit drinks, or candy (–0.43 servings; *P*=.001), but not both [20]. Furthermore, only 1 study within this review used a similar methodology of assessment based on lunchbox photography and observation to estimate serving size [20]. The study found no significant effect on the nutritional quality of food brought from home, which might have been due to a lack of power or a result of the complex information dissemination pathway of their intervention that involved sending messages to parents via newsletters and lessons delivered to children. To target behavior change, our research team conducted extensive formative assessment including mapping barriers and consulting with key stakeholders to co-design the SWAP IT using a theoretical framework. The multicomponent program also dually targeted both the school, via guidelines and curriculum, and parents, via direct messages, which may explain the favorable intervention effect.

To improve health at a population level, interventions shown to be effective under research conditions need to be scaled up to reach a large proportion of the population [46]. Few school-based behavioral interventions have been suggested to be suitable for large-scale dissemination, as they require expertise and resources not readily available within schools and often use high-intensity delivery modes, such as face-to-face training [20]. The use of digital delivery modes overcome many of these barriers; however, poor adoption and ongoing engagement with new apps or websites, for example, are often an impediment to population-level reach and improved health gains [47]. To address this, the SWAP IT intervention adopted a multicomponent intervention design that addressed many of the existing limitations [20]. Incorporating the SWAP IT behavioral intervention components as a complement into an existing school communication app that had already been adopted by schools and downloaded by parents was undertaken to overcome the challenges of both population-level reach and digital engagement. A similar approach, in which a nutrition intervention was embedded into an existing online school canteen ordering system, also resulted in a significant intervention effect on energy, sugar, and fat [48]. This suggests that embedding digital interventions within existing systems supported by additional behavior change strategies may be superior to developing and implementing new digital health interventions alone.

Although a reduction in energy from discretionary foods of 600 kJ per week may appear small at an individual level, at a population level, it has the potential to lower the risk of individuals being overweight or obese, result in a gain of health-adjusted life years, and make a significant contribution toward savings in health care costs [49]. Given its potential reach, with 86% of students taking a packed lunch on a daily basis [9] and 90% of those students packing at least 1 serving of discretionary food in their lunchbox [14], this intervention has the potential to immensely shift the consumption of discretionary foods. Further, as 60% to 70% [50] of schools in NSW Australia and the United States, respectively, already using school communication apps, interventions such as SWAP IT have the potential to reach millions of parents on a daily basis. Further investigation evaluating the cost-effectiveness and implementation process of the SWAP IT intervention is needed to confirm if the SWAP IT intervention warrants large scale dissemination. Future research should focus on developing strategies that maximize the adoption or uptake of the SWAP IT intervention by schools at scale and methods for sustaining school engagement to continue the impact on parent behavior change.

The results of this trial should be interpreted within the context of its strengths and limitations. Study strengths include the experimental hybrid design, with randomized controlled trials being considered the gold standard for evaluating causal effects of interventions. The SWAP IT trial was also developed using behavior change theory and used direct observation and validated tools to assess lunchbox contents, which strengthened the ability of the study to accurately measure the true impact of the study outcomes. Although the effect size of the SWAP IT effectiveness trial was smaller than that of the previous pilot [16], the significant results were replicated, indicating that, pending further evaluation exploring the implementation outcomes and cost effectiveness, the intervention warrants consideration for large-scale dissemination. However, a number of limitations should be considered. The trial had a lower than anticipated participation and consent rate from schools and particularly parents, with only 41.90% of parents consenting to participate in the lunchbox observations. Upon enquiry, we believe this is primarily due to the measurement component, in which lunchbox observations might have been considered an encroachment on privacy [51], given that the acceptability of the intervention for schools and parents was high at 84% [16]. The intervention was also multicomponent, and isolating the impact of each individual strategy was not possible in this trial. Although the large-scale dissemination of pushed messages to parents via the app is feasible and has high fidelity, implementation of school-level strategies may require additional support. This trial had a follow-up period of 6 months, and the long-term sustainability of the intervention in both schools and with parents is unknown. Further investigation is warranted to ensure the intervention has an ongoing desirable impact on lunchbox behavior.

The SWAP IT intervention presents an effective digital behavior change solution to a large and long-standing public health problem of a high consumption of discretionary foods by children while at school. Given the significant impact on lunchbox food energy that has been demonstrated by the previous pilot trial and replicated in this effectiveness trial at a larger scale, the intervention provides an attractive option to policy makers to complement existing public health programs targeting the school nutrition environment. Following further evaluation to determine its implementation process, outcomes, and cost-effectiveness, models to further scale up and maximize the adoption of SWAP IT will ensure that a public health benefit can be realized.

## Appendix

Multimedia Appendix 1 Using the Behaviour Change Wheel process to map barriers to packing healthy lunchboxes with identified intervention functions and suitable behaviour change techniques (BCTs).

Multimedia Appendix 2 Food and drink items packed in lunchboxes.

Multimedia Appendix 3 CONSORT-eHEALTH checklist (V 1.6.1).

## Abbreviations

CONSORT: Consolidated Standards of Reporting Trials
NSW: New South Wales
SEIFA: Socio-Economic Index for Areas
SFC: School Food Checklist
WHO: World Health Organization
