# Supplementary material for: A Multicomponent mHealth-Based Intervention (SWAP IT) to Decrease the Consumption of Discretionary Foods Packed in School Lunchboxes: Type I Effectiveness–Implementation Hybrid Cluster Randomized Controlled Trial
Source: J Med Internet Res. 2021 Jun 24;23(6):e25256. doi: 10.2196/25256 (PMC8277365; doi:10.2196/25256)
Supplement: Multimedia Appendix 1 [file jmir_v23i6e25256_app1.docx]

**Multimedia Appendix 1**

Table 1. Using the behaviour change wheel process to map barriers to packing healthy lunchboxes with identified intervention functions and suitable behaviour change techniques (BCTs)

| **Barrier Identified** | **Description** | **Strategies / Intervention Components** | **Intervention functions** | **How (BCTs used)** |
| --- | --- | --- | --- | --- |
| Child Preference | Packing what children like to (and will) eat. | Push notifications including images:   - Fussy eating - Increasing vegetables - Involving children in packing the lunchbox | Education  Persuasion | 1.1 Goal setting (behaviour)?  3.1 Social support (unspecified)  4.1 Instruction on how to perform a behaviour  5.1 Information about health consequences  7.1 Prompt/cue  8.2 Behaviour substitution  13.1 Identification of self as role model |
|  |  | Website content:   - Tips for fussy eater - Swap options | Education  Persuasion | 4.1 Instruction on how to perform a behaviour  5.1 Information about health consequences |
|  |  | Video:   - Kid’s choice - Healthy Tastes Good | Modelling | 6.1 Demonstration of the behaviour |
|  |  | Stickers | Environment restructuring | 7.1 Prompts/cues  12.1 Adding objects to the environment |
|  |  | Parent brochure:   - Ideas for fussy eaters | Education  Persuasion | 4.1 Instruction on how to perform the behaviour |
|  |  | Classroom flipcharts | Education  Persuasion | 4.1 Instruction on how to perform a behaviour  6.1 Demonstration of the behaviour |
| Knowledge and skills | The knowledge and skills to purchase and prepare healthy foods. | Push notifications:   - What a healthy lunchbox consists of - Benefits of healthy lunchboxes - Swaps in the lunchbox - Supermarket list | Education  Persuasion | 2.3 Self-monitoring of behaviour?  4.1 Instruction on how to perform a behaviour  5.1 Information about health consequences  8.2 Behaviour substitution |
|  |  | Website content:   - Definition of everyday and sometimes - Swap options | Education  Persuasion | 4.1 Instruction on how to perform the behaviour  5.1 Information about health consequences |
|  |  | Video:   - Monday to Friday | Modelling | 6.1 Demonstration of the behaviour |
|  |  | Parent brochure:   - Lunchbox facts - What an everyday lunchbox is made up of - Swaps | Education  Persuasion | 4.1 Instruction on how to perform the behaviour  5.1 Information on health consequences |
| Cost | The cost of purchasing healthy food. | Push notification:   - Healthy doesn’t = expensive | Education  Persuasion | 1.4 Action planning?  4.1 Instruction on how to perform a behaviour  8.2 Behaviour substitution |
|  |  | Website content:   - Tips to save money | Education  Persuasion | 4.1 Instruction on how to perform the behaviour |
|  |  | Video:   - The cost of healthy living | Modelling | 6.1 Demonstration of the behaviour |
|  |  | Parent brochure:   - Tips to save money | Education  Persuasion | 4.1 Instruction on how to perform the behaviour |
| Time | The time to prepare healthy foods. Looking for the convenience of a packaged product. | Push notification:   - Quick everyday lunchboxes | Education  Persuasion | 4.1 Instruction on how to perform a behaviour  8.2 Behaviour substitution |
|  |  | Website content:   - Tips to save time | Education  Persuasion | 4.1 Instruction on how to perform a behaviour |
|  |  | Video:   - Everyday foods quick and easy | Modelling | 6.1 Demonstration of the behaviour |
|  |  | Parent brochure:   - Tips to save time | Education  Persuasion | 4.1 Instruction on how to perform the behaviour |
| Food Safety | Hesitation to pack refrigerated everyday foods (e.g. dairy) due to concerns that food will not be safe when consumed. | Push notification | Education  Persuasion | 4.1 Instruction on how to perform the behaviour |
|  |  | Website content | Education  Persuasion | 4.1 Instruction on how to perform the behaviour  5.1 Information about health consequences |
|  |  | Parent brochure | Education  Persuasion | 4.1 Instruction on how to perform the behaviour |
|  |  | Ice brick provision | Environment restructuring | 7.1 Prompts/Cues  12. 1 Adding objects to the environment |
