# Supplementary material for: A Multicomponent mHealth-Based Intervention (SWAP IT) to Decrease the Consumption of Discretionary Foods Packed in School Lunchboxes: Type I Effectiveness–Implementation Hybrid Cluster Randomized Controlled Trial
Source: J Med Internet Res. 2021 Jun 24;23(6):e25256. doi: 10.2196/25256 (PMC8277365; doi:10.2196/25256)
Supplement: Multimedia Appendix 2 [file jmir_v23i6e25256_app2.docx]

**Multimedia Appendix 2**

**Table 1. Food and drink items packed in lunchboxes.**

| **SFC food or drink category** | **n (% of lunchboxes item)** |
| --- | --- |
| Main |  |
| - Bread* | 1947 (81.40) |
| - Leftovers/mixed dishes* | 109 (4.56) |
| - Fast food | 66 (2.76) |
| Snacks |  |
| - Fruit* | 1992 (83.28) |
| - Muesli and/or fruit bars | 1483 (62.00) |
| - Savoury biscuits | 586 (24.50) |
| - Chocolate biscuits | 520 (21.74) |
| - Vegetables* | 485 (20.28) |
| - Crisps | 463 (19.36) |
| - Extruded snacks | 429 (17.93) |
| - Cheese, eggs, dried fruit, nuts* | 373 (15.59) |
| - Yoghurt* | 354 (14.80) |
| - Sweet biscuits | 329 (13.75) |
| - Dips* | 327 (13.67) |
| - Chocolates and lollies | 295 (12.33) |
| - Rice or water crackers* | 293 (12.25) |
| - Cakes | 259 (10.83) |
| - Popcorn* | 205 (8.57) |
| - Rice cakes/corn thin* | 166 (6.94) |
| - Muffins | 152 (6.35) |
| - Noodles | 103 (4.31) |
| - Crispbreads* | 87 (3.64) |
| - Pretzels | 75 (3.14) |
| - Slices | 67 (2.80) |
| - Processed meat | 62 (2.59) |
| - ‘Everyday’ buns* | 44 (1.84) |
| - Fruit jelly | 31 (1.30) |
| - Leftovers/mixed dishes as a snack* | 31 (1.30) |
| - Dairy dessert | 30 (1.25) |
| - Butter popcorn | 29 (1.21) |
| - Bread* | 24 (1.00) |
| - Fast food snacks | 23 (0.96) |
| - Pikelets* | 22 (0.92) |
| - Donut | 20 (0.84) |
| - Custards* | 16 (0.67) |
| - Sometimes miscellaneous | 16 (0.67) |
| - Sometimes buns | 13 (0.54) |
| - Sauce | 13 (0.54) |
| - Tuna* | 13 (0.54) |
| - Refined cereals | 12 (0.50) |
| - Baked beans or legumes* | 11 (0.46) |
| - Pastries | 11 (0.46) |
| - Cheese and bacon roll | 10 (0.42) |
| - Wholegrain cereals* | 6 (0.25) |
| - Scone* | 3 (0.13) |
| Drinks |  |
| - Water* | 1483 (83.28) |
| - Juice or cordial | 230 (9.62) |
| - Fortified milk drink (e.g. breakfast drinks)* | 31 (1.30) |
| - Milk (full fat, reduced fat, flavoured)* | 25 (1.05) |
| - Soft drink | 2 (0.08) |

* Items classified as an ‘everyday’ food or drink
